# AI Hesitancy and Acceptability—Perceptions of AI Chatbots for Chronic Health Management and Long COVID Support: Survey Study

**DOI:** 10.2196/51086

**Published:** 2024-07-23

**Authors:** Philip Fei Wu, Charlotte Summers, Arjun Panesar, Amit Kaura, Li Zhang

**Affiliations:** 1 School of Business and Management, Royal Holloway, University of London, Egham, United Kingdom; 2 DDM Health, Coventry, United Kingdom; 3 Warwick Medical School, University of Warwick, Coventry, United Kingdom; 4 Imperial College Healthcare NHS Trust, London, United Kingdom; 5 Department of Computer Science, Royal Holloway, University of London, Egham, United Kingdom

**Keywords:** AI hesitancy, chatbot, long COVID, diabetes, chronic disease management, technology acceptance, post–COVID-19 condition, artificial intelligence

## Abstract

**Background:**

Artificial intelligence (AI) chatbots have the potential to assist individuals with chronic health conditions by providing tailored information, monitoring symptoms, and offering mental health support. Despite their potential benefits, research on public attitudes toward health care chatbots is still limited. To effectively support individuals with long-term health conditions like long COVID (or post–COVID-19 condition), it is crucial to understand their perspectives and preferences regarding the use of AI chatbots.

**Objective:**

This study has two main objectives: (1) provide insights into AI chatbot acceptance among people with chronic health conditions, particularly adults older than 55 years and (2) explore the perceptions of using AI chatbots for health self-management and long COVID support.

**Methods:**

A web-based survey study was conducted between January and March 2023, specifically targeting individuals with diabetes and other chronic conditions. This particular population was chosen due to their potential awareness and ability to self-manage their condition. The survey aimed to capture data at multiple intervals, taking into consideration the public launch of ChatGPT, which could have potentially impacted public opinions during the project timeline. The survey received 1310 clicks and garnered 900 responses, resulting in a total of 888 usable data points.

**Results:**

Although past experience with chatbots (*P*<.001, 95% CI .110-.302) and online information seeking (*P*<.001, 95% CI .039-.084) are strong indicators of respondents’ future adoption of health chatbots, they are in general skeptical or unsure about the use of AI chatbots for health care purposes. Less than one-third of the respondents (n=203, 30.1%) indicated that they were likely to use a health chatbot in the next 12 months if available. Most were uncertain about a chatbot’s capability to provide accurate medical advice. However, people seemed more receptive to using voice-based chatbots for mental well-being, health data collection, and analysis. Half of the respondents with long COVID showed interest in using emotionally intelligent chatbots.

**Conclusions:**

AI hesitancy is not uniform across all health domains and user groups. Despite persistent AI hesitancy, there are promising opportunities for chatbots to offer support for chronic conditions in areas of lifestyle enhancement and mental well-being, potentially through voice-based user interfaces.

## Introduction

Artificial intelligence (AI) chatbots are programs designed to simulate human conversations and provide tailored responses to users’ questions and concerns. Chatbots can provide a range of services, including appointment scheduling, medication reminders, and various types of health support. AI chatbots have the potential to support individuals with chronic health conditions by providing tailored information and resources, monitoring symptoms, and offering emotional support [1]. While there are some limitations to chatbots’ use, they could be a valuable tool for individuals with long-term conditions such as long COVID (or post–COVID-19 condition) [2] and those living in remote or rural areas [3,4].

Researchers have long investigated the use of chatbots in managing various chronic illnesses. For example, past studies have documented how chatbots improved medication adherence rates of patients with breast cancer [5]; enhanced the quality of life for people with type 2 diabetes [6]; reduced the severity of panic disorder symptoms [7]; and helped health care professionals, patients with asthma, and their family members build collaborative relationships [8]. Several systematic literature reviews on the topic suggest that conversational agents are generally effective in supporting the self-management of chronic conditions, particularly for mental health [9-11]. Hence, “empathic” chatbots that demonstrate “emotional intelligence” seem particularly relevant and useful. Although some researchers use the term emotional intelligence to denote a chatbot’s ability to express a full range of human sentiments (positive and negative) [12], in the health care context, an emotionally intelligent chatbot usually refers to a conversational agent being able to recognize and respond to emotions a user expresses in their interaction and that “uses evidence-based self-help practices such as CBT, DBT, motivational interviewing, positive behavior support, behavioral reinforcement, mindfulness, and guided microactions and tools to encourage users to build emotional resilience skills” [13].

Despite the potential benefits of AI chatbots in health care, empirical research on public attitudes toward health care chatbots is still in its early stages [14]. Some early studies have suggested that users are generally positive about the use of AI chatbots [15]. For example, Bickmore et al [16] found that participants were generally satisfied with a health care chatbot that provided them with medication reminders and lifestyle advice. Similarly, a study by Crutzen et al [17] found that a health promotion chatbot targeting adolescents was used intensively and evaluated positively, especially in comparison with information lines and search engines. However, recent research has also highlighted many challenges associated with the use of AI chatbots in health care. There are concerns about the ability of chatbots to understand complex medical issues and provide accurate advice [11,18]. Patients and medical researchers alike were skeptical about the use of chatbots for mental health support, citing concerns about the lack of empathetic communication and the potential for the chatbot to misunderstand their emotional states [18,19].

Research on voice-based chatbots for health management is also in its infancy. Medical professionals’ views on voice-based chatbots echo views on text-based chatbots in terms of the technology’s potential and limitations. A 2-round Delphi study [20] surveying experts on the future of voice-controlled AI agents in health care anticipates significant technological development and increased user trust. The study focused on how voice-controlled agents could support health care professionals through applications like remote real-time interviews with patients, hands-free instructions for medical staff, and communication between staff and patients. However, the authors concluded that chatbots are not expected to outperform or replace human health care workers despite a more intuitive speech interaction.

A systematic review conducted in 2020 examining the literature on voice-based conversational agents for chronic health conditions only found 12 papers [21]. In another scoping review conducted in 2021, only 4 studies among 32 reviewed focused on voice-based chatbots in health care [11]. The consensus in the literature seems to be that the technology shows feasibility and acceptability for managing chronic diseases, but more research is still needed on their real-world efficacy. Importantly, these literature reviews highlight several limitations in the literature such as small sample sizes, questionable sample compositions (healthy or convenience samples instead of samples of patient groups), and not controlling for participants’ previous experience with voice-based intelligent agents.

In summary, AI chatbots have the potential to provide targeted support and improve the management of various chronic diseases, but only if they are designed to meet the specific needs and preferences of their users. It is essential to understand target users’ perspectives, preferences, and experiences of using chatbots for health purposes so that chatbot solutions address the needs of their intended users. Individuals with long-term health conditions often face complex challenges that require ongoing tailored support, while the extant research on using chatbots for health care support provides limited insights into the acceptance (or resistance) among people with chronic conditions. To address the limitations identified in previous studies [11,21], this study gathered a large sample of people with chronic conditions and delved into their past experiences of and future preferences for interacting with AI chatbots.

## Methods

### Overview

A web-based survey study was conducted between January 1 and March 31, 2023, targeting the diabetes.co.uk user population. The site is the largest web-based community of people with diabetes in Europe with hundreds of thousands of registered users [22]. We chose to target this population because our previous research collaborations showed the community’s wide awareness and practice of using technological solutions to self-manage their long-term health conditions (people with diabetes often experience other chronic conditions) [23,24]. For the survey, we defined AI chatbots broadly as computer programs designed to interact in humanlike conversation, and referred to Alexa and Siri on smart devices as examples of AI chatbots. As ChatGPT was launched on November 30, 2022, and quickly gained popularity, public understanding and opinions of chatbots might have changed during our project timeline. Hence, we aimed to capture the survey responses at multiple intervals. Several social media advertisements with the survey link were posted in January, February, and March 2023 on the Facebook page for diabetes.co.uk and clicked on by 1310 people.

As part of a research project funded by an Innovate UK grant, the survey was administered through the Qualtrics software by the digital health company leading the project. The purpose of the study and a consent form were presented on the landing page of the web survey. After the survey was closed, we exported response data from Qualtrics to SPSS v.28 (IBM Corp) for a quality check, data recoding, and variable labeling. We carefully examined the initial data set to remove duplicate records (mainly generated in the survey setup and testing process) and poor-quality responses such as those who completed (or abandoned) the survey in less than 120 seconds. The final data set for analysis contained 888 records. We also used SPSS to assign numeric values to all the nominal variables in the survey (eg, 1=male, 2=female). After the data set was cleaned, we exported it as a .csv file to R version 4.2.3 (The R Foundation for Statistical Computing) for frequency, crosstabulation, and regression tests.

The survey contained 30 questions: 24 closed questions, 2 open questions, and 4 demographic questions. Participant consent was provided at the start of the survey before completion. Quantitative information (closed and multiple-choice questions) was collected on four topic areas: (1) demographic characteristics; (2) long COVID symptoms and clinical diagnoses; (3) health apps, websites, and chatbot use; and (4) opinions about chatbots. The majority of the questions were adapted from the digital health literature (eg, [14,25]). To address the two research objectives, we first asked questions on general attitude and acceptance toward chatbots, such as “How familiar are you with AI chatbots?” and “How likely are you to use a health chatbot within the next 12 months if available?”; then we focus on questions on using chatbots in specific health management scenarios, such as “Do you think chatbots have the capability of delivering accurate medical advice?” and “Would you like a chatbot to understand your stress levels and emotional states?” We also included questions on widely cited factors in the literature that might affect chatbot adoption such as trust and privacy.

### Ethical Considerations

The survey instrument, along with other details of the methodology, was approved by the Royal Holloway, University of London (Full-Review-3509-2022-11-18-13-35-UATM024). The participants were presented with a consent page before starting the web-based questionnaire. The purpose and the method of the research were briefly explained, along with an informed consent form asking participants to confirm that they were 18 years or older and that they were voluntarily taking part in the study. The survey did not collect personally identifiable information. The IP addresses were monitored to prevent multiple entries from the same computer. However, all IP addresses were removed from the data when the survey closed. Each survey response was assigned a unique ID, and the encrypted data were stored in the United Kingdom on Microsoft Azure servers. No compensation was provided to participants for completing the survey.

## Results

### Demographic Characteristics

Of the 888 individuals who started the survey, 729 (82.1%) responded to the sex question, of which 471 (64.6%) were female, 252 (34.6%) were male, and the remaining 6 (0.8%) were nonbinary/third sex or “prefer not to say.” Of the 741 respondents who provided their age, 556 (75%) were 55 years or older, with a median age of 63 (IQR 55-70) years. The sample consisted predominantly of White (n=466,64.2%) individuals; other ethnicities were represented in smaller percentages and scattered across different categories of ethnicity (eg, Indian and Pakistani: n=25, 3.5%; Black n=7, 1.2%).

In relation to chronic health conditions, almost half of the 888 respondents reported having type 2 diabetes (n=437, 49.2%). Table 1 provides an overview of the top 10 chronic conditions identified in the survey responses, plus long COVID. Of the 740 individuals who responded to the question “Would you describe yourself as having Long COVID?” 170 (23%) answered yes. While a majority of the respondents utilized health apps (n=500, 73.5%), a much smaller portion (n=259, 38.1%) made use of voice-assisted apps or devices like Amazon Alexa.

**Table 1.** Most common chronic health conditions (n=888).

Frequency rank	Condition	Frequency, n (%)
1	Type 2 diabetes	437 (49.2)
2	High blood pressure/hypertension	330 (37.2)
3	Alzheimer’s disease	240 (27.0)
4	Long COVID	170 (19.1)
5	Arthritis	195 (22.0)
6	Allergies	187 (21.1)
7	Anxiety	157 (17.7)
8	Depression	137 (15.4)
9	Asthma	114 (12.8)
10	Type 1 diabetes	106 (11.9)
11	Chronic pain	102 (11.5)

### Attitudes Toward Health Chatbots

Although the survey was conducted at a time when ChatGPT was beginning to receive wide public attention, a significant number of respondents were “not familiar at all” (n=272, 40.5%) or only “slightly familiar” (n=175, 26%) with AI chatbots. There was an overall hesitancy about using a health chatbot, with less than one-third of respondents (n=203, 30.1%) indicating that they were “somewhat likely” or “very likely” to use a health chatbot in the next 12 months if available.

However, a deeper dive into the survey data reveals a more nuanced picture. There seems to be a great deal of uncertainty among people about AI chatbots’ capability of providing accurate medical advice. When asked if they believe chatbots have the capability of providing accurate medical advice, 396 of 677 (58.5%) respondents answered “unsure,” while only 77 (11.4%) answered “yes” and 204 (30.1%) chose “no.”

On the other hand, people seem to be more open to the idea of chatbots supporting mental well-being: 272 (40.2%) would like a chatbot to understand their stress levels and emotional states, 211 (31.2%) were unsure, and 194 (28.7%) indicated no interest. A further cross-tabulation and *χ*^*2*^ analysis using the chisq function in R suggests that people with long COVID in our sample were more likely to be interested in an emotionally intelligent chatbot than those without long COVID (n=673; *χ*^2^_2_=13.73; *P*=.001), although nearly one-third of the former group were still “unsure” (Table 2).

**Table 2.** Cross-tabulation: long COVID chatbot understands stress and emotion.

			Long COVID?		Total (n=673)
			Yes (n=162)	No (n=511)	
**Would you like a chatbot to understand your stress and emotional states?**					
	Yes		81 (50.0)	190 (37.2)	271 (40.3)
	No		29 (17.9)	164 (32.1)	193 (28.7)
	Unsure		52 (32.1)	157 (30.7)	209 (31.1)
	Did not respond		—^a^	—	215 (24.2^b^)

a Not applicable.

b Percentage based on the 888 total respondents.

This “chatbot hesitancy” is also evident when comparing people’s preferences of a bot and a real person in various health scenarios. Overall, our survey respondents overwhelmingly prefer to speak to a real person rather than a bot about physical and mental health. However, people seem to not mind speaking with a bot about nutrition and sleep as much or letting it collect symptom data and conduct some preliminary analysis as indicated in Table 3.

**Table 3.** People’s preferences of a bot and a real person in various health scenarios.

Would you prefer a bot or a real person when...	Bot, n (%)	Person, n (%)	Don’t mind, n (%)	Did not respond, n (%)^a^
Speaking about general health (n=600)	18 (3.0)	484 (80.7)	98 (16.3)	288 (32.4)
Speaking about mental health (n=596)	26 (4.4)	483 (81.0)	87 (14.6)	292 (32.9)
Speaking about sleep (n=595)	46 (7.7)	370 (62.2)	179 (30.1)	293 (33.0)
Speaking about nutrition (n=599)	64 (10.7)	343 (57.3)	192 (32.1)	289 (32.5)
Collecting symptoms (n=590)	63 (10.7)	270 (45.8)	257 (43.6)	298 (33.6)
Conducting preliminary analysis (n=596)	72 (12.1)	314 (52.7)	210 (35.2)	292 (32.9)

a Percentages in this column are based on the total 888 respondents.

Consistent with the observations above, an encouraging sign for health chatbot developers is that people are willing to try voice-based health chatbots despite the overwhelming hesitance toward bots. Of 679 respondents, 309 (45.5%) expressed willingness to use a voice-based chatbot to record their health symptoms on a mobile device, and 278 (41.1%) would let their voice be analyzed to diagnose health problems. When asked if they would like to trial a voice-based health chatbot that the research team is developing, 364 of 560 (65%) respondents answered “yes,” as illustrated in Table 4.

**Table 4.** Attitude toward voice-based health chatbot.

	Yes, n (%)	No, n (%)	Unsure, n (%)	Didn’t respond, n (%^a^)
Would you use your voice to record health symptoms on a mobile device? (n=679)	309 (45.5)	182 (26.8)	188 (27.7)	209 (23.5)
Would you use an app that analyzes your voice to diagnose potential health problems? (n=679)	278 (41.1)	158 (23.3)	241 (35.6)	209 (23.5)
Would you like to trial the voice-based health chatbot we are developing? (n=560)	364 (65.0)	196 (35.0)	—^b^	328 (36.9)

a Percentages in this column are based on the total 888 respondents.

b Option not provided.

### Factors Predicting Health Chatbot Adoption

We ran linear regression analyses in R to explore factors that could predict individuals’ likelihood to use a health chatbot in the next 12 months. We categorized the variables into three groups: demographic, experience, and attitudinal. Table 5 presents our findings.

**Table 5.** Predictors analysis using regression. Likelihood of adopting a health chatbot in the next 12 months (n=485).^a^

		β	SE	*P* value	95% CI
**Demographic**					
	Age	–.075	.005	.06	–.021 to .000
	Sex	*.078* ^*b*^	*.097*	*.03*	*.039 to .460*
	Long COVID?	–.064	.115	.08	–.428 to .023
**Experience**					
	Familiarity with AI^c^ chatbot	*.169*	*.048*	*<.001*	*.110 to .302*
	Frequency of online health information seeking	*.198*	*.012*	*<.001*	*.039 to .084*
**Attitudinal**					
	Comfortable with reporting symptoms to health chatbot	*.228*	*.060*	*<.001*	*.142 to .377*
	Worry about privacy using health chatbot	–.032	.042	.40	–.128 to .042
	Trust health chatbot	*.255*	*.069*	*<.001*	*.219 to .489*

a Likelihood of adoption was measured on a 5-point Likert scale (1=extremely unlikely and 5=extremely likely). *R*2=0.388.

b Italics indicate statistical significance.

c AI: artificial intelligence.

We considered *P* values <.05 as statistically significant in our regression analysis results. The results showed that age (β=–.075; *P*=.06) and long COVID status (β=−.064; *P*=.08) have little to do with participants’ tendency to use a health chatbot. Sex, dummy-coded as male=1 and female=2 with other sex categories excluded from this analysis due to a small number in each of the categories, seems to have a marginal effect (β=.078; *P*=.03), with female respondents potentially being more inclined to adopt a health chatbot than male respondents. Past experience with an AI chatbot (“familiarity with AI chatbots”; β=.169; *P*<.001) and online health information-seeking frequency (aggregated frequencies across Google, social media, and professional health sites; β=.198; *P*<.001) show strong associations with chatbot adoption likelihood. Similarly, two attitudinal items measuring a person’s comfort in outlining symptoms to a health chatbot (β=.228; *P*<.001) and their trust in a chatbot for advice (β=.255; *P*<.001) also strongly predicted their likelihood of adopting a health chatbot. Interestingly, privacy concerns, despite being widely reported in the academic literature as a deterrent to chatbot or virtual assistant adoption [20,26], did not seem to affect the likelihood of survey respondents adopting a health chatbot (β=−.032; *P*=.40).

## Discussion

### Principal Findings

The survey results summarized above present a nuanced portrayal of public attitudes toward health care chatbots. The findings indicate that trust continues to be a crucial element in predicting people’s inclination to embrace health chatbots, aligning with prior research on user acceptance of digital health technologies [11,27,28]. It seems that for our sample of predominantly female adults older than 55 years, most of them do not trust a chatbot to provide accurate diagnosis and professional medical advice. This echoes findings in previous studies [11,29] that while patients were generally receptive to the use of AI chatbots in health care, they had concerns about the accuracy of information provided and the ability of chatbots to understand complex medical issues. Although past experience with chatbots and online information seeking are strong indicators of respondents’ future adoption of health chatbots, they are in general skeptical or unsure about the use of AI chatbots for health care purposes. Because of this “AI hesitancy” [14], it is unsurprising that most people show an overall preference for a real person (clinician) over a chatbot in health care encounters.

On the other hand, this study contributes fresh insights into overcoming AI hesitancy and the potential use of AI chatbots in supporting long-term health conditions like long COVID. A key finding from the survey is that AI hesitancy is not uniform across all health domains and user groups. A significant proportion of survey respondents expressed willingness to engage with a health chatbot regarding nutrition and sleep, as well as allowing it to collect symptom data. Furthermore, although doubts about the medical capabilities of AI chatbots persist, people are more receptive to utilizing them for stress detection and emotional enhancement. Notably, individuals with long COVID in our sample exhibited a particular interest in emotionally intelligent chatbots, highlighting the mental health needs of those with long COVID and the potential of using conversational agents as an intervention [30]. It is also surprising that privacy concerns did not correlate with the likelihood of health chatbot adoption in our study, a finding contrary to conclusions in many previous studies [20,31]. Leveraging these positive attitudes toward AI chatbots could enhance public familiarity and increase the likelihood of chatbot adoption for health care purposes, as evidenced by our regression analysis. For instance, an AI chatbot focusing on lifestyle or emotional well-being could pave the way for broader acceptance of health chatbots that are reliable and highly personalized [32].

This research also provides preliminary insights into the potential of voice-based interaction with health chatbots. Despite the popularity of voice-based AI agents such as Siri and Alexa on smart devices, there are only a handful of academic studies on the public’s attitude toward an alternative voice-based interface for health chatbots [11,21]. Traditional text-based chatbots on mobile phones present challenges to older adults in terms of vision and dexterity, as typing on a smartphone can be difficult and the screen size is often too small for them [33]. From the technology acceptance research, we understand that the usability of a health chatbot plays a role in its perceived usefulness [32]. Therefore, the relatively high acceptability and enthusiasm toward voice-based health chatbots expressed in our survey responses indicate a potential avenue for reaching a wider, often neglected population of adults older than 55 years. In addition to the usability benefits, voice input can be captured and analyzed for symptom tracking and medical diagnosis [34], complementing other data inputs to enable a more accurate assessment of the user’s health.

### Limitations

It is noteworthy that this study had several limitations. The use of a web-based survey for the empirical study introduces the potential for response bias. The sample primarily consisted of female adults older than 55 years with diabetes and other long-term health conditions, rather than representing the general population. As this population may have been more attuned to ongoing health concerns, they might have been more prone to reporting long COVID symptoms, potentially explaining the significantly higher proportion of those with long COVID in our sample compared to the national data from the National Health Service. The UK COVID-19 Infection National Survey (monthly, terminated in March 2023) reported that around 3% of the UK population were experiencing symptoms 4 weeks after they first had COVID-19 [35].

As cross-sectional survey research, this study is unable to provide a deep understanding of attitudes and opinions. For example, we do not know exactly why people are more receptive to voice-based AI chatbots aside from an educated guess that a voice interface may be easier to use and more natural than typed text for adults older than 55 years. Future research based on in-depth interviews or experimental methods might help unpack these user attitudes reported in the survey and identify possible causal factors.

### Conclusion

With the rapid development of AI and chatbot technologies, the utilization of chatbots in health care is primed for substantial expansion in the forthcoming years. The potential benefits offered by these technologies, such as enhanced health care accessibility [4,36], cost reduction [15], and improved patient outcomes [37], are too substantial to disregard. However, health care providers and technology developers must acknowledge AI hesitancy [14] among patients and ensure the inclusive and effective utilization of AI chatbots.

This study contributes valuable insights into the acceptability of health chatbots among a population identified as requiring continuous long-term health care support, such as individuals at high risk for conditions like long COVID [38]. First, we augment the evidence in the literature that there exists a general skepticism toward health chatbots among people with chronic diseases. Second, notwithstanding this persistent AI hesitancy, we found that individuals are more receptive toward chatbots supporting lifestyle enhancement than those aiding disease diagnosis and health care management. Third, compared to other subgroups in the study, those with long COVID were more amenable to using chatbots for emotional support. Lastly, while popular AI chatbots on the market are text-based, this study demonstrates that individuals with chronic conditions exhibit a high interest in voice-based conversational agents despite their general AI hesitancy. Moving forward, it is paramount to continue exploring the potential applications of health chatbots in addressing the unique needs of specific patient populations, including those with chronic health conditions.

## Abbreviations

AI: artificial intelligence
